# Clinical Impact of Obesity on Postoperative Outcomes of Patients With Thyroid Cancer Undergoing Thyroidectomy: A 5‐Year Retrospective Analysis From the US National Inpatient Sample

**DOI:** 10.1002/cam4.70335

**Published:** 2024-10-17

**Authors:** Yue Chen, Jiewen Jin, Pengyuan Zhang, Runyi Ye, Chuimian Zeng, Yilin Zhang, Junxin Chen, Hai Li, Haipeng Xiao, Yanbing Li, Hongyu Guan

**Affiliations:** ^1^ Department of Endocrinology and Diabetes Center The First Affiliated Hospital of Sun Yat‐sen University Guangzhou China; ^2^ Department of Breast Surgery The First Affiliated Hospital of Sun Yat‐sen University Guangzhou Guangdong China; ^3^ Zhongshan School of Medicine Sun Yat‐sen University Guangzhou China

## Abstract

**Background:**

The clinical impact of obesity on postoperative outcomes of patients undergoing thyroidectomy remains controversial.

**Methods:**

Patients aged ≥ 18 years who were diagnosed with thyroid malignancy and underwent thyroidectomy between 2016 and 2020 were included, and divided into two groups: patients with body mass index (BMI) < 30 kg/m^2^ and those with BMI ≥ 30 kg/m^2^. Patients in the obese group were then subdivided into four groups: Group 1 (BMI 30.0–34.9 kg/m^2^), Group 2 (BMI 35.0–39.9 kg/m^2^), Group 3 (BMI 40.0–44.9 kg/m^2^), and Group 4 (BMI ≥ 45.0 kg/m^2^) to evaluate the association between degree of obesity and clinical outcomes. We performed propensity score matching, compared outcome variables between the groups, and conducted adjusted multivariate logistic regression analyses of postoperative outcomes.

**Results:**

A total of 6778 patients diagnosed with thyroid cancer who underwent thyroidectomy were screened, of whom 1299 (19.2%) patients were obese. Patients in the obese group had higher total hospital charges (*p* < 0.001) and an increased risk of overall postoperative complications (34.7% vs. 30.5%, *p* = 0.023). Specifically, patients in the obese group had increased odds of respiratory complication (adjusted odds ratio (aOR) 1.66, 95% confidence interval (CI) [1.26–2.19]), acute renal failure (aOR 1.87, 95% CI [1.13–3.09]), and wound complication (aOR 2.77, 95% CI [1.21–6.37]) than those in the non‐obese group. Moreover, trend tests showed that the risks of unfavorable discharge, infection, acute renal failure, and respiratory complication all exhibited an upward trend with increased BMI.

**Conclusion:**

Obesity is associated with an increased risk of postoperative complications in patients with thyroid cancer undergoing thyroidectomy. This finding suggests that obese patients should be treated with more caution during postoperative recovery.

## Graphical abstract

In this retrospective study, using a large sample of data from the National Inpatient Sample, we compared the differences in short‐term postoperative outcomes between obese and non‐obese patients who underwent thyroidectomy for thyroid malignancies in a cohort of over 6700 patients.

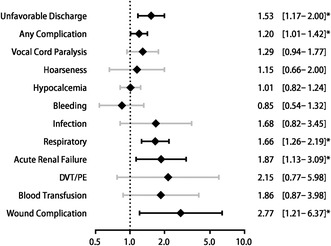

## 1 Introduction

Defined as abnormal or excessive fat accumulation that may impair health and diagnosed at a body mass index (BMI) of ≥ 30 kg/m^2^, as defined by the World Health Organization (WHO), obesity is currently a major public health burden in both developed and developing countries and poses great challenges to the global population [1]. During the past decades, the global prevalence of overweight and obesity has increased dramatically [2]. As recent WHO global estimates show, worldwide obesity has nearly tripled since 1975 [1]. In 2016, 39% of adults, numerically more than 1.9 billion, were overweight, of whom over 650 million were obese [1]. There is unanimous consensus that obesity is a risk factor for a wide range of chronic diseases, including hypertension, dyslipidemia, diabetes mellitus type 2, cardiovascular disease, non‐alcoholic fatty liver disease, polycystic ovarian syndrome, osteoarthritis, Alzheimer's disease, and various cancers. Thus, obesity leads to decreased quality of life and life expectancy [3, 4]. Several organizations, including the World Obesity Federation (WOF) and the American and Canadian Medical Associations, have noted the importance of obesity as a distinct chronic progressive disease instead of simply considering it as a risk factor for other diseases [5]. Besides the health risks it entails at the individual level, the obesity pandemic may create a heavy health and economic burden for human society [6, 7].

Thyroid cancer is the most common endocrine malignancy with a rapidly escalating incidence worldwide [8]. As Global Cancer Statistics 2020 demonstrated, there were 586,202 cases of thyroid cancer worldwide in 2020, and it ranked in the 9th and 5th places for total incidence and incidence in women, respectively [9]. Despite its relatively indolent nature and low mortality rate, thyroid cancer still accounted for an estimated 44,000 deaths worldwide among both the sexes and a mortality rate of 0.43 cases per 100,000 person‐years [9]. Although radioactive iodine (RAI) therapy serves as an alternative and supplement, surgical removal remains the mainstay of treatment for differentiated thyroid cancers [10].

Accumulating evidence have shown the effects of obesity on patients undergoing surgical procedures for a wide range of diseases, and obesity has been found to be associated with more postoperative complications [11, 12]. Generally, obesity markedly increases the risk of wound infection, causes more bleeding, and prolongs the duration of the surgery [13]. Successful completion of thyroid surgery requires careful attention to intraoperative management and monitoring of the recurrent laryngeal nerve (RLN) and the preservation of the parathyroid glands, which may become more challenging in patients with obesity [14].

However, the effect of obesity on postoperative outcomes in patients undergoing thyroidectomy remains controversial. For instance, Buerba et al. investigated 18,825 patients who underwent thyroidectomy and found that high BMI was associated with longer operative time and increased wound complication [15]. It has been reported that BMI is an independent risk factor for locoregional events in patients with macropapillary thyroid cancer undergoing thyroid operation [16]. Other studies concluded that obesity was not a risk factor for thyroidectomy. Studies performed by Finel et al. and Farag et al. showed that there was no significant association between obesity and adverse outcomes of thyroid surgery [17, 18].

Thus, this study aimed to evaluate the risk posed by obesity in patients with thyroid cancer undergoing thyroidectomy. We compared the local‐specific complications and short‐term surgical outcomes of thyroidectomy for thyroid cancer between obese and non‐obese groups in a large cohort of over 6000 patients from a nationwide US database.

## 2 Materials and Methods

### 2.1 Database Description

All patient data were obtained from the National Inpatient Sample (NIS) between 2016 and 2020. The NIS is part of a family of databases developed and administered by the Healthcare Cost and Utilization Project (HCUP). The NIS is the largest publicly available all‐payer US inpatient care database, which contains data regarding approximately 7–8 million hospitalizations each year [19]. The data is a 20%‐stratified sample of discharges from US community hospitals. The data elements within the database include primary and secondary diagnoses, primary and secondary procedures, admission type, patient demographics, expected payment source, length of hospital stay, and hospital characteristics. The International Classification of Diseases, Tenth Revision, Clinical Modification/Procedure Coding System (ICD‐10‐CM/PCS) codes have been used in the database since 2016.

### 2.2 Ethics Statement

As the NIS is a de‐identified publicly available database, the study was exempted from Institutional Review Board (IRB) approval and informed consent.

### 2.3 Study Design

**FIGURE 1:**
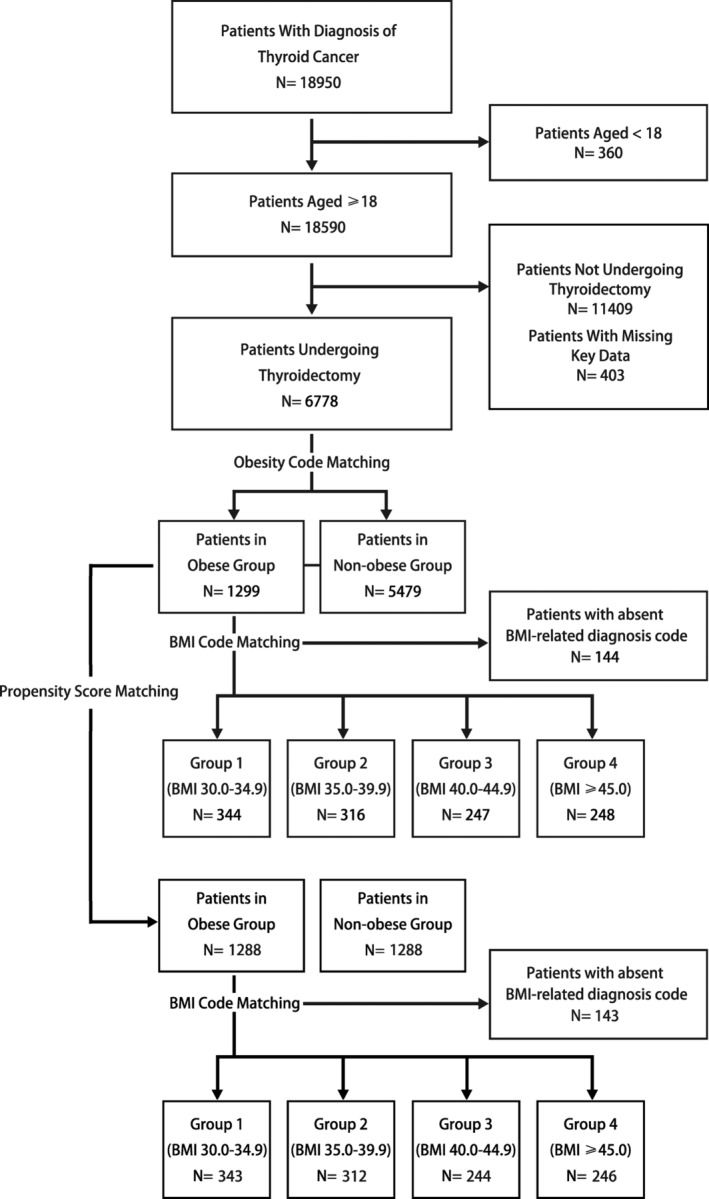
Flow chart of the patient population selection and division.

Based on the ICD‐10‐CM/PCS, we identified all patients aged ≥ 18 years who were diagnosed with thyroid cancer and underwent thyroidectomy between 2016 and 2020. Records missing key data were excluded from the analysis. According to the WHO definition, obesity was diagnosed as a BMI ≥ 30 kg/m^2^. The identified patients with thyroid cancer were divided into two groups: the non‐obese group with BMI < 30 kg/m^2^ and the obese group with BMI ≥ 30 kg/m^2^. The patients in the obese group were then subdivided into four groups: Group 1 (BMI 30.0–34.9 kg/m^2^), Group 2 (BMI 35.0–39.9 kg/m^2^), Group 3 (BMI 40.0–44.9 kg/m^2^), and Group 4 (BMI ≥ 45.0 kg/m^2^). The workflow in Figure 1 shows the selection and subdivision of patients included in this study. Details of the ICD‐10‐CM/PCS codes utilized are shown in Table S1 [20, 21].

The length of stay, total hospital charges, unfavorable discharge status, and surgical or postoperative complications were assessed as the main dependent variables. Surgical and postoperative complications included bleeding, wound complication (wound disruption or intraoperative complication), deep vein thrombosis (DVT)/pulmonary embolism (PE), respiratory complication, infection, blood transfusion, and acute renal failure. Potential significant complications of thyroidectomy, namely hypoparathyroidism and RLN injury, were also assessed by evaluating hypocalcemia, vocal cord paralysis, and hoarseness. Details of the ICD‐10 codes used are summarized in Table S1 [22, 23]. Length of stay and total hospital charges were calculated continuously. Considering the patients' discharge outcomes, transfer to a short‐term hospital, home health care, or other transfers including a skilled nursing facility, intermediate care, and another type of facility, were defined as unfavorable discharge.

Patient and hospital variables including patient age, race/ethnicity, sex, expected primary payer, median household income, national quartile for patient ZIP code, patient location, smoking habit, admission type, surgical type (total, unilateral, or partial), common comorbidities, and hospital characteristics were assessed. Age was continuously assessed. Surgical type and comorbidities were identified using the ICD‐10 diagnostic codes [23]. Hospital characteristics included hospital bed size, hospital location, and teaching status (rural, urban non‐teaching, or urban teaching).

### 2.4 Statistical Analyses

Continuous variables are presented as medians and interquartile ranges owing to skewed data, while categorical variables are shown as proportions. To compare the non‐obese and obese groups, continuous variables were tested using the Mann–Whitney *U* test, and categorical variables were tested using Pearson's chi‐squared test with or without Yates' continuity correction. Fisher's exact test was performed when approximations of the chi‐squared tests were inadequate. For comparison between the non‐obese group and the four BMI subgroups, Kruskal–Wallis test, Pearson's chi‐squared test, Fisher's test, and Cochran–Armitage trend test were performed.

Given the substantial difference in chronic comorbidity burden between the obese and non‐obese groups, propensity score matching was performed to reduce possible confounding factors and induce a balance between the two groups when estimating the effect of obesity. We established a logistic regression model incorporating age and comorbidities to calculate the propensity score. Then, one‐to‐one greedy nearest neighbor matching without replacement was performed by employing calipers with a width of 0.25 of the standard deviation of the logit of the estimated propensity score. The matching procedure was done using R package “MatchIt” [24]. The standardized mean differences (SMD) of comorbidity covariates used in the propensity score modeling were calculated to evaluate the balance achieved after matching. A threshold of SMD < 0.1 (10%) for a given variable was considered to achieve balance [25].

To assess the impact of obesity and BMI on postoperative complications, multivariate logistic regression analysis of the matched cohort was performed. Patients in the non‐obese group were used as the reference group in the multivariate logistic regression analyses of the four BMI subgroups. In both multivariate logistic regression analyses conducted for clinical outcomes (whether comparing two or five groups), adjustments were made for baseline variables that were not included in the propensity score modeling.

The statistical software packages SAS (version 9.4) and R (version 4.3.1) were used to perform statistical analyses. Statistical significance was set at a two‐sided *p* < 0.05.

## 3 Results

### 3.1 Patient and Hospital Characteristics

A total of 6778 patients aged ≥ 18 years who were diagnosed with thyroid cancer and underwent thyroidectomy were screened from the NIS database (2016–2020); of them, 1299 (19.2%) patients were obese and 5479 (80.8%) were non‐obese.

The baseline demographics, clinical, and hospital characteristics of patients in these two groups are summarized in Table 1. Significant differences were observed between the two groups in race/ethnicity, income quartile, expected primary payer, hospital region, depression, hypertension, congestive heart failure, chronic pulmonary disease, liver disease, renal disease, and diabetes mellitus. Patients who were obese were more likely to be Black (9.3% vs. 6.0%) or Hispanic (20.6% vs. 18.1%) and were more frequently covered by Medicaid (18.2% vs. 14.9%) than non‐obese patients. Additionally, patients in the obese group had a heavier burden of chronic comorbidities than non‐obese patients.

**TABLE 1:** Characteristics of hospitals and patients before and after matching in obese and non‐obese group undergoing thyroidectomy for thyroid cancer, National Inpatient Sample 2016–2020.

Demographic characteristics	Unmatched cohort				Matched cohort		
	Obese group, *n* = 1299 (%)	Non‐obese group, *n* = 5479 (%)	*p*	SMD	Obese group, *n* = 1288 (%)	Non‐obese group, *n* = 1288 (%)	*p*
**Age (year, median, IQR)**	52 (41–63)	54 (39–67)	0.165	0.040	52 (41–63)	54 (42–65)	0.110
Race/ethnicity			**< 0.001**				**< 0.001**
White	62.7	61.5		0.024	62.6	59.0	
Black	9.3	6.0		0.115	9.3	6.8	
Hispanic	20.6	18.1		0.062	20.7	18.9	
Other^a^	7.5	14.5		0.266	7.5	15.2	
Gender			0.774				0.284
Female	65.7	66.2		0.009	65.6	63.6	
Male	34.3	33.8		0.009	34.4	36.4	
Income quartile by zip code			**0.005**				0.525
1st	24.5	23.1		0.033	24.4	25.2	
2nd	25.5	23.1		0.054	25.6	24.5	
3rd	26.3	25.2		0.025	26.0	24.4	
4th	23.8	28.7		0.115	24.0	26.0	
Primary expected payer			**0.015**				0.542
Medicare	27.9	30.5		0.059	27.7	29.8	
Medicaid	18.2	14.9		0.087	18.2	16.7	
Private	48.0	49.0		0.020	48.1	47.1	
Other^b^	5.9	5.6		0.012	6.0	6.4	
Admission type			0.326				0.149
Elective	86.4	85.3		0.031	86.3	84.2	
Non‐elective	13.6	14.7		0.031	13.7	15.8	
Procedure type			0.612				0.426
Total thyroidectomy	64.9	64.8		0.003	64.9	66.4	
Unilateral thyroidectomy	30.0	30.8		0.016	30.0	29.6	
Partial thyroidectomy	5.1	4.5		0.027	5.0	4.0	
Hospital bed size			0.261				0.283
Small	11.1	12.7		0.053	11.1	12.9	
Medium	20.2	20.1		0.003	20.2	21.0	
Large	68.7	67.1		0.033	68.7	66.1	
Hospital location/teaching status			0.438				0.533
Rural	2.2	2.3		0.007	2.2	2.3	
Urban non‐teaching	10.3	11.5		0.040	10.2	11.6	
Urban teaching	87.5	86.2		0.040	87.6	86.1	
Hospital region			< **0.001**				**0.004**
Northeast	22.9	25.5		0.060	22.8	26.6	
Midwest	21.6	15.3		0.152	21.4	17.0	
South	26.2	30.2		0.091	26.2	29.0	
West	29.3	29.1		0.006	29.6	27.4	
Chronic comorbidity							
Smoking habit	8.2	7.2	0.212	0.037	8.3	6.6	0.099
Depression	13.2	6.8	< **0.001**	0.190	12.7	11.1	0.223
Hypertension	58.7	39.2	< **0.001**	0.396	58.4	60.0	0.400
Congestive heart failure	6.3	2.7	< **0.001**	0.150	6.1	6.1	1.000
Chronic pulmonary disease	18.2	11.0	< **0.001**	0.187	17.8	16.8	0.498
Liver disease	2.8	1.7	**0.007**	0.069	2.8	3.0	0.814
Diabetes mellitus	30.4	15.3	< **0.001**	0.329	29.8	31.5	0.347
Renal disease	7.5	4.5	< **0.001**	0.111	7.5	8.4	0.423
Metastatic solid tumor	43.3	44.2	0.539	0.019	43.2	43.0	0.905

^a^ Includes Asian or Pacific Islander, Native American, and other.

^b^ Includes self‐pay, no charge, and others. Statistical significance was defined as a two‐sided *p*‐value < 0.05. The values in bold in the tables represent results that meet this threshold for statistical significance.

Among patients in the obese group, 144 had no BMI‐related diagnoses and were not considered for the BMI subgroup analysis. Patients with multiple distinct BMI diagnoses were grouped based on the earliest BMI diagnoses. Table 2 shows the baseline patient demographics of the non‐obese group and four obese subgroups stratified by BMI. Statistically significant differences were observed in age, race/ethnicity, sex, income quartile, expected primary payer, hospital region, hypertension, depression, congestive heart failure, chronic pulmonary disease, liver disease, renal disease, and diabetes mellitus. Patients in Group 4 were younger (48 [40–58]) while patients in Group 1 tended to be older (57 [44–67]). Regarding chronic comorbidities, most diseases, including congestive heart failure, chronic pulmonary disease, and diabetes mellitus, were increasingly prevalent with higher BMI.

**TABLE 2:** Characteristics of Hospitals and patients in non‐obese group and the four obese subgroups stratified by BMI ranges undergoing thyroidectomy for thyroid cancer, National Inpatient Sample 2016–2020.

Demographic characteristics	Non‐obese group (*n* = 5479)	BMI 30.0–34.9 (*n* = 344)	BMI 35.0–39.9 (*n* = 316)	BMI 40.0–44.9 (*n* = 247)	BMI ≥ 45.0 (*n* = 248)	*p*
Age (year, median, IQR)	54 (39–67)	57 (44–67)	53 (41–63)	51 (40–61)	48 (40–58)	< **0.001**
Race/ethnicity						< **0.001**
White	61.5	59.9	60.4	66.8	66.1	
Black	6.0	7.8	9.5	11.3	9.7	
Hispanic	18.1	22.7	25.9	15.4	16.5	
Other^a^	14.5	9.6	4.1	6.5	7.7	
Gender						**0.030**
Female	66.2	59.6	67.1	71.3	69.4	
Male	33.8	40.4	32.9	28.7	30.6	
Income quartile by zip code						**0.021**
1st	23.1	24.1	21.8	27.9	28.6	
2nd	23.1	23.3	26.6	24.7	26.6	
3rd	25.2	26.5	29.7	24.7	23.8	
4th	28.7	26.2	21.8	22.7	21.0	
Primary expected payer						**0.024**
Medicare	30.5	30.8	23.7	25.5	25.8	
Medicaid	14.9	17.7	14.6	20.6	18.5	
Private	49.0	47.1	53.5	48.6	48.4	
Other^b^	5.6	4.4	8.2	5.3	7.3	
Admission type						0.174
Elective	85.3	88.4	89.2	84.2	85.1	
Non‐elective	14.7	11.6	10.8	15.8	14.9	
Procedure type						0.591
Total thyroidectomy	64.8	66.9	66.5	59.1	67.3	
Unilateral thyroidectomy	30.8	27.9	28.5	35.6	28.2	
Partial thyroidectomy	4.5	5.2	5.1	5.3	4.4	
Hospital bed size						0.724
Small	12.7	10.2	10.1	10.9	12.9	
Medium	20.1	18.6	19.9	19.8	19.4	
Large	67.1	71.2	69.9	69.2	67.7	
Hospital location/teaching status						0.260
Rural	2.3	2.0	1.9	2.0	2.8	
Urban non‐teaching	11.5	9.3	13.6	10.1	6.5	
Urban teaching	86.2	88.7	84.5	87.9	90.7	
Hospital region						<**0.001**
Northeast	25.5	23.8	22.8	18.6	22.6	
Midwest	15.3	18.6	18.4	24.7	29.4	
South	30.2	20.1	27.5	31.2	29.4	
West	29.1	37.5	31.3	25.5	18.5	
Chronic comorbidities						
Smoking habit	7.2	10.8	7.6	7.7	5.2	0.106
Depression	6.8	11.9	13.6	10.9	15.3	**< 0.001**
Hypertension	39.2	55.5	53.8	57.9	67.3	**< 0.001**
Congestive heart failure	2.7	3.5	5.1	8.5	8.9	**< 0.001**
Chronic pulmonary disease	11.0	14.0	15.2	18.6	26.2	**< 0.001**
Liver disease	1.7	2.0	4.1	4.0	1.2	**0.006**
Diabetes mellitus	15.3	26.2	31.0	31.6	34.7	**< 0.001**
Renal disease	4.5	6.4	6.0	8.9	6.0	**0.011**
Metastatic solid tumor	44.2	43.6	47.8	38.9	43.1	0.327

^a^ Includes Asian or Pacific Islander, Native American, and other.

^b^ Includes self‐pay, no charge, and others. Statistical significance was defined as a two‐sided *p*‐value < 0.05. The values in bold in the tables represent results that meet this threshold for statistical significance.

**FIGURE 2:**
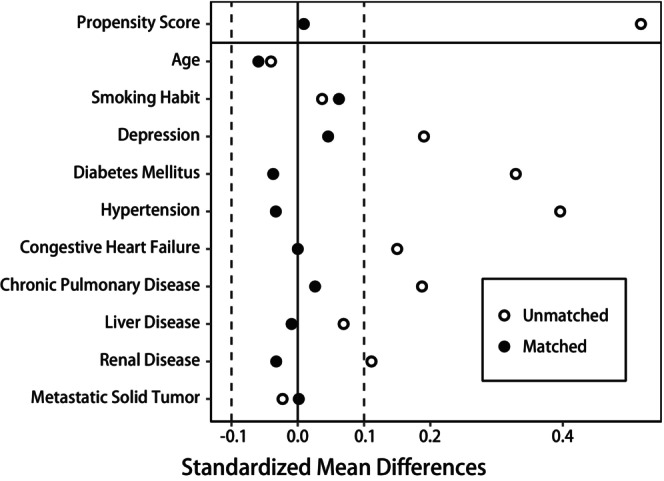
Love plot showing comparison of standardized mean differences in propensity score, age, and comorbidities between obese and non‐obese patients before and after propensity score matching.

We obtained 1288 pairs from the entire cohort after one‐to‐one propensity score matching. As shown in Figure 2, all covariates included in the propensity score model were well‐balanced, with all standardized mean differences below 0.1. The baseline characteristics before and after matching are summarized in Table 1.

### 3.2 Clinical Outcomes

The inpatient outcomes of the non‐obese and obese groups after thyroidectomy are presented in Table 3. Higher values and proportions of these outcomes were observed in the obese group. Patients in the obese group had higher total hospital charges (59,460 [38,283–96,486] vs. 53,513 [35,412–85,797], *p* < 0.001) than those in the non‐obese group. Although statistically significant, there was no substantial disparity in terms of length of stay (2 [1–4] vs. 2 [1–3], *p* < 0.001). The unfavorable discharge and overall complication rates in the obese group were 12.5% and 34.7%, respectively, which were significantly higher than those in the non‐obese group (8.9% [*p* = 0.003] and 30.5% [*p* = 0.023], respectively]. Patients in the obese group had higher rates of respiratory complication (11.8% vs. 7.9%, *p* < 0.001), acute renal failure (3.6% vs. 2.0%, *p* = 0.013), and wound complication (1.6% vs. 0.6%, *p* = 0.015).

**TABLE 3:** Post‐matching clinical outcomes of patients in obese and non‐obese group undergoing thyroidectomy for thyroid cancer, National Inpatient Sample 2016–2020.

Clinical outcomes	Obese group, *n* = 1288 (%)	Non‐obese group, *n* = 1288 (%)	*p*
Length of stay (days, median, IQR)	2 (1–4)	2 (1–3)	**< 0.001**
Adjusted hospital charges ($, median, IQR)	59,460 (38,283–96,486)	53,513 (35,412–85,797)	**< 0.001**
Unfavorable discharge	12.5	8.9	**0.003**
Any complication	34.7	30.5	**0.023**
Vocal cord paralysis	7.6	6.1	0.139
Hoarseness	2.3	1.9	0.409
Hypocalcemia	18.4	17.9	0.759
Bleeding	3.0	3.5	0.435
Infection	1.6	1.0	0.167
Respiratory	11.8	7.9	**< 0.001**
Acute renal failure	3.6	2.0	**0.013**
DVT/PE	1.0	0.5	0.107
Blood transfusion	1.6	0.9	0.104
Wound complication	1.6	0.6	**0.015**

*Note:* Mann–Whitney *U* tests were performed for length of stay and adjusted hospital charges. Pearson's chi‐squared tests with or without Yates' continuity correction and Fisher's exact tests were performed for postoperative complications. Statistical significance was defined as a two‐sided *p*‐value < 0.05. The values in bold in the tables represent results that meet this threshold for statistical significance.

Table 4 shows the inpatient outcomes of the subgroups stratified by the BMI ranges. There was no substantial difference in the length of hospital stay among the subgroups. Patients in Group 3 had the lowest median of hospital charges (53,355 [35,941–89,654]) among the obese subgroups. Intriguingly, the results of the Cochran–Armitage trend test demonstrated that the rates of unfavorable discharge, infection, respiratory complication, and acute renal failure showed an upward trend as BMI increased.

**TABLE 4:** Post‐matching clinical outcomes of patients undergoing thyroidectomy for thyroid cancer in non‐obese group and the four obese subgroups stratified by BMI ranges, National inpatient sample 2016–2020.

Complications	Non‐obese group (*n* = 1288)	BMI 30.0–34.9 (*n* = 343)	BMI 35.0–39.9 (*n* = 312)	BMI 40.0–44.9 (*n* = 244)	BMI ≥ 45.0 (*n* = 246)	*p*
Length of stay (d, median, IQR)	2 (1–3)	2 (1–3)	2 (1–4)	2 (1–4)	2 (1–4)	**0.016**
Adjusted hospital charges ($, median, IQR)	53,513 (35,412–85,797)	61,060 (39,676–97,504)	60,623 (40,964–97,796)	53,355 (35,941–89,654)	62,188 (37,399–97,065)	**< 0.001**
Unfavorable discharge	8.9	9.0	10.9	15.6	13.0	**0.002**
Any complication	30.5	32.1	35.9	33.6	34.6	0.074
Vocal cord paralysis	6.1	7.9	7.4	7.8	5.7	0.669
Hoarseness	1.9	2.9	1.6	1.6	3.3	0.433
Hypocalcemia	17.9	16.0	17.9	16.8	21.1	0.489
Bleeding	3.5	3.8	2.2	3.7	2.0	0.284
Infection	1.0	0.9	1.0	1.6	2.8	**0.037**
Respiratory	7.9	8.7	11.5	13.1	12.6	**< 0.001**
Acute renal failure	2.0	1.2	4.2	4.1	4.1	**0.005**
DVT/PE	0.5	0.6	0.3	1.6	1.2	0.063
Blood transfusion	0.9	2.9	1.0	0.4	0.8	0.701
Wound complication	0.6	2.6	0.6	0.4	2.0	0.226

*Note:* Kruskal–Wallis tests were performed for length of stay and adjusted hospital charges. Cochran–Armitage trend tests were performed for postoperative complications. Statistical significance was defined as a two‐sided *p*‐value < 0.05. The values in bold in the tables represent results that meet this threshold for statistical significance.

### 3.3 Multivariate Logistic Regression

**FIGURE 3:**
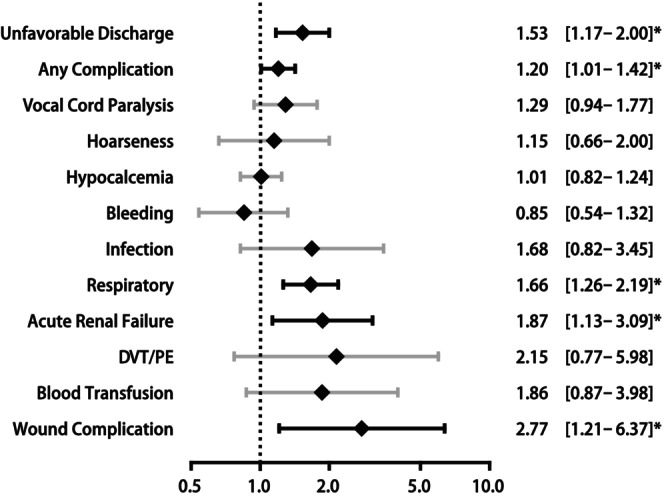
Forest plot of adjusted odds ratios comparing clinical outcomes in obese group versus. non‐obese group undergoing thyroidectomy for thyroid cancer after matching. Models were adjusted for race/ethnicity, gender, income quartile by zip code, primary expected payer, admission type, procedure type, hospital bedsize, hospital location/teaching status and hospital region. *p < 0.05.

After matching and adjusting, patients in the obese group had approximately 2.8 times the odds of wound complication (adjusted odds ratio (aOR) 2.77, 95% confidence interval (CI) [1.21–6.37], *p* = 0.016) and approximately 1.9 times the odds of acute renal failure (aOR 1.87, 95% CI [1.13–3.09], *p* = 0.015) compared to patients in the non‐obese group. Significant increases were also observed for respiratory complication (aOR 1.66, 95% CI [1.26–2.19], *p* < 0.001), unfavorable discharge (aOR 1.53, 95% CI [1.17–2.00], *p* = 0.002), and any complication (aOR 1.20, 95% CI [1.01–1.42], *p* = 0.036) (Table S2, Figure 3).

**FIGURE 4:**
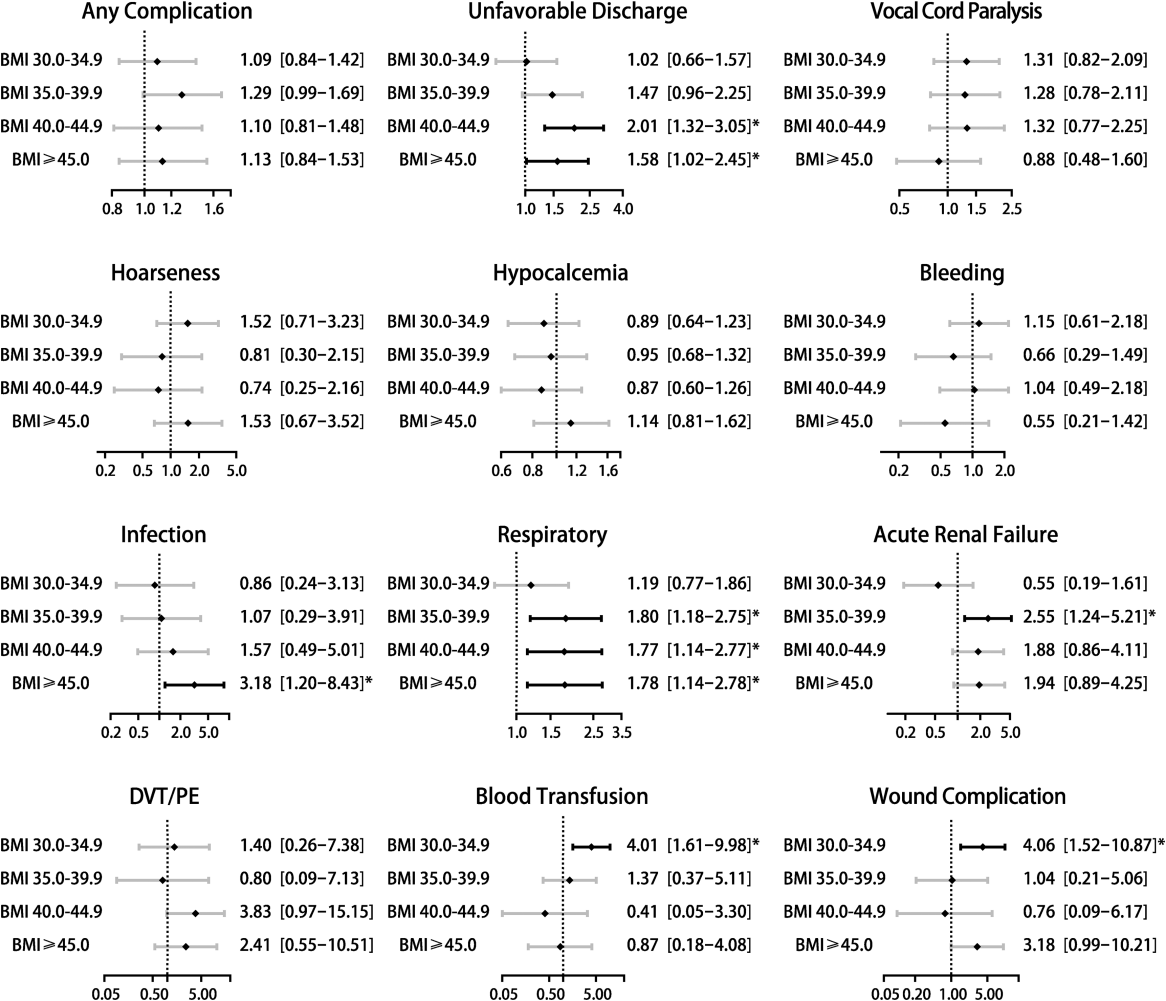
Forest plot of adjusted odds ratios comparing clinical outcomes in obese group vs. the four obese subgroups stratified by BMI ranges undergoing thyroidectomy for thyroid cancer after matching. Models were adjusted for race/ethnicity, gender, income quartile by zip code, primary expected payer, admission type, procedure type, hospital bedsize, hospital location/teaching status and hospital region. *p < 0.05.

The forest plots of the adjusted odds ratio of postoperative outcomes grouped by BMI ranges are shown in Figure 4. Considering the non‐obese group as the reference, patients in Group 1 had 4 times the risk of blood transfusion (aOR 4.01, 95% CI [1.61–9.98], *p* = 0.003) and 4 times the risk of wound complication (aOR 4.06, 95% CI [1.52–10.87], *p* = 0.005). Patients in Group 2 had 1.8 times the risk of experiencing respiratory complication (aOR 1.80, 95% CI [1.18–2.75], *p* = 0.006) and approximately 2.6 times the risk of acute renal failure (aOR 2.55, 95% CI [1.24–5.21], *p* = 0.011). Patients in Group 3 had approximately double the risk of having an unfavorable discharge (aOR 2.01, 95% CI [1.32–3.05], *p* = 0.001), and 1.8 times the risk of experiencing respiratory complication (aOR 1.77, 95% CI [1.14–2.77], *p* = 0.012). Patients in Group 4 had 3.2 times the risk of infection (aOR 3.18, 95% CI [1.20–8.43], *p* = 0.020), 1.8 times the risk of respiratory complication (aOR 1.78, 95% CI [1.14–2.78], *p* = 0.011), and 1.6 times the risk of having an unfavorable discharge (aOR 1.58, 95% CI [1.02–2.45], *p* = 0.040) (Table S3, Figure 3).

## 4 Discussion

In this retrospective study, using a large sample of data from the NIS, we compared the differences in short‐term postoperative outcomes between obese and non‐obese patients who underwent thyroidectomy for thyroid malignancies in a cohort of over 6700 patients. After one‐to‐one propensity score matching, we obtained 1288 pairs that were well‐matched. In addition, nearly 1300 obese patients were subdivided into four groups based on their BMI ranges to evaluate the association between the degree of obesity and clinical outcomes. Our results showed that obesity was associated with worse clinical outcomes, including higher total hospital charges, increased likelihood of being discharged in an unfavorable state, and higher risks of several postoperative complications. Trend tests showed that the rates of unfavorable discharge, infection, respiratory complication, and acute renal failure exhibited an upward trend as BMI increased. After adjusting for covariates, a significantly increased risk of unfavorable discharge, any complication, acute renal failure, and respiratory and wound complication was observed in the obese group.

Major complications that can potentially occur after thyroidectomy, such as hypocalcemia, vocal cord paralysis, hoarseness, and hematoma, are of great concern. Our results showed no correlation between these main complications and BMI. A prospective study conducted by Finel et al. showed that although operative time was significantly increased in patients with a BMI of ≥ 25 kg/m^2^, no statistically significant difference was observed in the occurrence of RLN palsy, hypoparathyroidism, and bleeding complication [17]. High BMI was not associated with hypocalcemia, RLN palsy, and postoperative pain, but was correlated with a longer duration of procedure in patients undergoing thyroidectomy [26]. It is likely because thyroidectomy is a superficial procedure that does not require a significant incision, and because the visceral and subcutaneous fat of the neck is thinner than that of the trunk. However, we observed that obesity was an independent risk factor for wound complication (wound disruption or intraoperative complication) (aOR = 2.77, *p* = 0.016). Patients in Group 1 had approximately 4 times (aOR = 4.06, *p* = 0.005) the risk of wound complication. The correlation between wound disruption and obesity has been well reported and can be explained by multiple mechanisms, including the anatomic features of adipose tissue, vascular insufficiency, and oxidative stress [27].

Patients with obesity present with general postoperative complications more often than non‐obese patients, including in thyroid surgery. Konishi et al. designed a restricted cubic spline (RCS) analysis to investigate the association between BMI and postoperative outcomes and found that obesity was significantly associated with postoperative length of stay, total hospital charges, duration of anesthesia, the occurrence of overall general complication, and respiratory complication [28]. A study by Buerba et al. indicated that patients with high BMI undergoing thyroidectomy were prone to longer operation time and more morbidity [15]. Consistent with these studies, we found that patients in the obese group paid more total in‐hospital charges compared to non‐obese patients (59,460 (38,283–96,486) vs. 53,513 (35,412–85,797), *p* < 0.001), were at a higher risk of respiratory complication than non‐obese patients (aOR 1.66, *p* < 0.001), and that these rates increased as BMI increased (*p* < 0.001). This corresponds with the previous study by Konishi et al. [28] Moreover, patients in Group 2, Group 3, and Group 4 exhibited approximately 1.8 times the risk of respiratory complication (Group 2: aOR = 1.80, *p* = 0.006; Group 3: aOR =1.77, *p* = 0.012; Group 4: aOR = 1.78, *p* = 0.011). The association between obesity and postoperative respiratory complication has been previously reported in several types of surgery [29, 30]. Obesity could lead to alterations in the pattern of breathing, increase respiratory resistance, and cause ventilation perfusion mismatch and gas exchange abnormalities [31, 32]. Adipose tissue could yield reduced lung volumes and lung compliance in obese patients, resulting in an increased risk of developing respiratory complication [33].

Our study showed that patients in the obese group were at an increased risk of infection, which agrees with a previous study by Jin et al. [34] In the present study, a trend test confirmed that the infection rate increased with increasing BMI (*p* = 0.037). Moreover, patients with a BMI of ≥ 45 kg/m^2^ exhibited a nearly three‐fold higher risk of infection (aOR = 3.18, *p* = 0.020); various mechanisms may be involved in the elevated risk of infection in such patients. Fat accumulation disrupts lymphoid tissue integrity, reduces antigen response, causes chronic inflammation, and leads to higher rates of comorbidities such as type 2 diabetes, all of which are likely to contribute to obesity‐induced immune dysfunction [35, 36]. Increased risk of infection in patients with obesity have been described previously in thyroidectomy and various other surgeries [37, 38]. Thus, these patients should be medically optimized during and after surgery to contain the potential infection.

In the present study, obesity was found to be an independent risk factor for postoperative acute renal failure (aOR = 1.87, *p* = 0.015). We observed an approximately two‐fold higher rate of acute renal failure in the obese group (3.6% vs. 2.0%, *p* = 0.013), and the rate of acute renal failure showed an increasing trend as BMI increased (*p* = 0.005). Patients in Group 2 had approximately 2.6 times (aOR = 2.55, *p* = 0.011) the risk of acute renal failure. The association between obesity and renal disease has been extensively reported [39, 40]. It could be explained by obesity‐caused hemodynamic changes, such as glomerular hyperperfusion and hyperfiltration, which may result in glomerular injuries [41, 42].

The NIS is the largest all‐payer inpatient care database in the United States. To our knowledge, no analysis of obese patients undergoing thyroidectomy for thyroid cancer has been performed using data from the NIS. With a sample size of nearly 6700, we were able to enhance the statistical power of our study and reduced the likelihood of sampling errors. In addition, through propensity score matching, we minimized potential biases caused by the existing comorbidity burden, thereby making our results robust. The NIS database includes data from a broad range of hospitals and patient demographics, and its national representativeness ensures that the results of our study can be generalized to a wider population of inpatients.

This study has several limitations. It was designed as a retrospective analysis, and the known inherent limitations of this type of analysis, namely, recall, misclassification, and selection bias, apply to this study. The length of hospital stay, which was not calculated from the day of surgery, might not precisely reflect postoperative outcomes because elective surgery might have been delayed for various reasons unrelated to patients' health status. Patient background characteristics such as pathological classification, cancer stage, and intraoperative device use, which might affect clinical outcomes, were not available in the NIS. Additionally, because the NIS provides no way to assess BMI, the subdivisions in this study depended on ICD‐10 codes, which might include misclassification or miscoding. Moreover, obesity‐related codes might have been missed from the patients' medical records, as obesity was neither the primary diagnosis nor the reason for admission in most of the cases. This may have resulted in an underestimation of the obese population. Few patients in the non‐obese group had a BMI‐related diagnosis; therefore, in the BMI subgroup analysis, we were unable to subdivide the non‐obese patients in the same manner as we did for the obese patients. Moreover, the NIS is not a longitudinal database, and patient data after discharge, including follow‐up, outpatient visits, and readmission data, are not available. Thus, we mainly focused on the postoperative outcomes that occurred during the patients' in‐hospital stay. Adverse outcomes that emerged after discharge and the readmission rates could not be assessed. Despite these limitations, this study provides a national perspective for future research.

## 5 Conclusions

Our analysis revealed that in patients undergoing thyroidectomy for thyroid malignancy, obesity is associated with an increased risk of adverse inpatient outcomes, including higher total hospital charges and increased general postoperative complications. These results suggest that patients with obesity should be treated with more caution during surgery and during postoperative recovery. Our results will be useful for optimal preoperative triage, improved operative risk stratification, and better discharge planning. They can also offer guidance to surgeons by providing appropriate informed consent and better postoperative care instructions to patients and their caretakers.

## Author Contributions

**Yue Chen:** data curation (lead), formal analysis (lead), methodology (lead), writing – original draft (lead). **Jiewen Jin:** data curation (lead), formal analysis (equal), funding acquisition (equal), methodology (lead), writing – original draft (equal). **Pengyuan Zhang:** data curation (supporting), investigation (equal), methodology (supporting). **Runyi Ye:** methodology (equal), resources (equal). **Chuimian Zeng:** data curation (supporting), writing – original draft (supporting). **Yilin Zhang:** investigation (supporting), methodology (supporting). **Junxin Chen:** formal analysis (equal). **Hai Li:** data curation (supporting), validation (equal). **Haipeng Xiao:** supervision (equal), writing – review and editing (equal). **Yanbing Li:** conceptualization (lead), project administration (lead), writing – review and editing (equal). **Hongyu Guan:** conceptualization (lead), funding acquisition (lead), project administration (lead), writing – review and editing (lead).

## Conflicts of Interest

The authors declare no conflicts of interest.

## Supporting information


Table S1.

## Data Availability

The data supporting the findings of this study are available at the National Inpatient Sample (NIS) database, https://hcup‐us.ahrq.gov/db/nation/nis/nisdbdocumentation.jsp.
