# Supplementary material for: Clinical Impact of Obesity on Postoperative Outcomes of Patients With Thyroid Cancer Undergoing Thyroidectomy: A 5‐Year Retrospective Analysis From the US National Inpatient Sample
Source: Cancer Med. 2024 Oct 17;13(19):e70335. doi: 10.1002/cam4.70335 (PMC11483747; doi:10.1002/cam4.70335)
Supplement: Supplementary file 1 — Table S1. [file CAM4-13-e70335-s001.docx]

| **Supplemental Table 1**  ICD-10 codes for defining obesity, obesity subgroups based on BMI ranges, thyroid cancer, thyroidectomy, metastasis, smoking habit, surgical type and postoperative complications. | |
| --- | --- |
| **Variable** | **ICD-10 codes** |
| Obesity | E66.01, E66.09, E66.1, E66.2, E66.8, E66.9, Z68.30-Z68.45 |
| Group 1(BMI 30.0-34.9) | Z68.30, Z68.31, Z68.32, Z68.33, Z68.34 |
| Group 2(BMI 35.0-39.9) | Z68.35, Z68.36, Z68.37, Z68.38, Z68.39 |
| Group 3(BMI 40.0-44.9) | Z68.41 |
| Group 4(BMI 45.0 or greater) | Z68.42, Z68.43, Z68.44, Z68.45 |
| Thyroid Cancer | C73, D09.3 |
| Total Thyroidectomy | 0GTK0ZZ, 0GTK4ZZ |
| Unilateral Thyroidectomy | 0GTG0ZZ, 0GTG4ZZ, 0GTH0ZZ, 0GTH4ZZ |
| Partial Thyroidectomy | 0GTJ0ZZ, 0GTJ4ZZ, 0GBG0ZX, 0GBG0ZZ, 0GBG3ZX, 0GBG3ZZ, 0GBG4ZX, 0GBG4ZZ, 0GBH0ZX, 0GBH0ZZ, 0GBH3ZX, 0GBH3ZZ, 0GBH4ZX, 0GBH4ZZ, 0GBJ0ZX, 0GBJ0ZZ, 0GBJ3ZX, 0GBJ3ZZ, 0GBJ4ZX, 0GBJ4ZZ |
| Smoking Habit | Z72.0, F17.200, F17.210 |
| Hypocalcemia | E83.51,E83.59 |
| Hoarseness | R47.1, R49.0, R49.1, R49.21, R49.22, R49.8, R49.9 |
| Vocal Cord Paralysis | J38.00, J38.01, J38.02 |
| Bleeding | E07.89, R57.1, R58, E36.01, E36.02, E89.810, E89.811, E89.820, E89.821, E89.822, E89.823, E89.89, L76.31, L76.32, 0G9K00Z, 0G9K0ZX, 0G9K0ZZ, 0G9K30Z, 0G9K3ZX, 0G9K3ZZ, 0G9K40Z, 0G9K4ZX, 0G9K4ZZ, 0GCK0ZZ, 0GCK3ZZ, 0GCK4ZZ, 0WJ60ZZ, 0WJ63ZZ, 0WJ64ZZ, 0WJ6XZZ, 0GJS0ZZ, 0GJS3ZZ, 0GJS4ZZ, 0GJK0ZZ, 0GJK3ZZ, 0GJK4ZZ, 0GWK00Z, 0GWK30Z, 0GWK40Z, 0GWKX0Z, 03LU0CZ, 03LU0DZ, 03LU0ZZ, 03LU3CZ, 03LU3DZ, 03LU3ZZ, 03LU4CZ, 03LU4DZ, 03LU4ZZ, 03LV0CZ, 03LV0DZ, 03LV0ZZ, 03LV3CZ, 03LV3DZ, 03LV3ZZ, 03LV4CZ, 03LV4DZ, 03LV4ZZ |
| Wound Complication | T81.30XA, T81.31XA, T81.32XA, T81.33XA, T81.4XXA, T81.83XA, E36.11, G97.49, L76.12, M96.821, T88.8XXA, T81.82XA, T81.89XA |
| Respiratory | J12.0, J12.1, J12.2, J12.3, J12.81, J12.82, J12.89, J12.9, J13, J14, J15.0, J15.1, J15.20, J15.211, J15.212, J15.29, J15.3, J15.4, J15.5, J15.6, J15.7, J15.8, J15.9, J16.0, J16.8, J17, J18.0, J18.1, J18.2, J18.8, J18.9, J69.0, J95.00, J95.01, J95.02, J95.03, J95.04, J95.09, J95.4, J95.5, J95.811, J95.812, J95.821, J95.822, J95.830, J95.831, J95.84, J95.850, J95.851, J95.859, J95.860, J95.861, J95.862, J95.863, J95.88, J95.89, J96.00, J96.01, J96.02, J96.90, J96.91, J96.92, J98.11, J98.19, J98.8 |
| Acute Renal Failure | N17.0, N17.1, N17.2, N17.8, N17.9 |
| DVT/PE | I26.01, I26.02, I26.09, I26.92, I26.99, I82.401, I82.402, I82.403, I82.409, I82.411, I82.412, I82.413, I82.419, I82.421, I82.422, I82.423, I82.429, I82.431, I82.432, I82.433, I82.439, I82.441, I82.442, I82.443, I82.449, I82.491, I82.492, I82.493, I82.499, I82.4Y1, I82.4Y2, I82.4Y3, I82.4Y9, I82.4Z1, I82.4Z2, I82.4Z3, I82.4Z9 |
| Blood Transfusion | 30230AZ, 30230C0, 30230G0, 30230G1, 30230G2, 30230G3, 30230G4, 30230H0, 30230H1, 30230J0, 30230J1, 30230K0, 30230K1, 30230L0, 30230L1, 30230M0, 30230M1, 30230N0, 30230N1, 30230P0, 30230P1, 30230Q0, 30230Q1, 30230R0, 30230R1, 30230S0, 30230S1, 30230T0, 30230T1, 30230U2, 30230U3, 30230U4, 30230V0, 30230V1, 30230W0, 30230W1, 30230X0, 30230X1, 30230X2, 30230X3,30230X4, 30230Y0, 30230Y1, 30230Y2, 30230Y3, 30230Y4, 30233AZ, 30233C0, 30233G0, 30233G1, 30233G2, 30233G3, 30233G4, 30233H0, 30233H1, 30233J0, 30233J1, 30233K0, 30233K1, 30233L0, 30233L1, 30233M0, 30233M1, 30233N0, 30233N1, 30233P0, 30233P1, 30233Q0, 30233Q1, 30233R0, 30233R1, 30233S0, 30233S1, 30233T0, 30233T1, 30233U2, 30233U3, 30233U4, 30233V0, 30233V1, 30233W0, 30233W1, 30233X0, 30233X1, 30233X2, 30233X3, 30233X4, 30233Y0, 30233Y1, 30233Y2, 30233Y3, 30233Y4, 30240AZ, 30240C0, 30240G0, 30240G1, 30240G2, 30240G3, 30240G4, 30240H0, 30240H1, 30240J0, 30240J1, 30240K0, 30240K1, 30240L0, 30240L1, 30240M0, 30240M1, 30240N0, 30240N1, 30240P0, 30240P1, 30240Q0, 30240Q1, 30240R0, 30240R1, 30240S0, 30240S1, 30240T0, 30240T1, 30240U2, 30240U3, 30240U4, 30240V0, 30240V1, 30240W0, 30240W1, 30240X0, 30240X1, 30240X2, 30240X3, 30240X4, 30240Y0, 30240Y1, 30240Y2, 30240Y3, 30240Y4, 30243AZ, 30243C0, 30243G0, 30243G1, 30243G2, 30243G3, 30243G4, 30243H0, 30243H1, 30243J0, 30243J1, 30243K0, 30243K1, 30243L0, 30243L1, 30243M0, 30243M1, 30243N0, 30243N1, 30243P0, 30243P1, 30243Q0, 30243Q1, 30243R0, 30243R1, 30243S0, 30243S1, 30243T0, 30243T1, 30243U2, 30243U3, 30243U4, 30243V0, 30243V1, 30243W0, 30243W1, 30243X0, 30243X1, 30243X2, 30243X3, 30243X4, 30243Y0, 30243Y1, 30243Y2, 30243Y3, 30243Y4, 30280B1, 30283B1 |
| Infection | A40.0, A40.1, A40.3, A40.8, A40.9, A41.01, A41.02, A41.1, A41.2, A41.3, A41.4, A41.50, A41.51, A4152, A4153, A4159, A4181, A4189, A419, T81.12XA, T81.40XA, T81.41XA, T81.42XA, T81.43XA, T81.44XA, T81.49XA, T81.4XXA, L02.11, L02.12, L02.13 |

| **Supplemental Table 2**  Post-matching logistic regression analysis of morbidity in patients in obese group vs non-obese group undergoing thyroidectomy for thyroid cancer, National Inpatient Sample 2016-2020. | | | |
| --- | --- | --- | --- |
|  | Adjusted Odds Ratio^a^ | 95%CI | P-value |
| Unfavorable Discharge | 1.53 | 1.17-2.00 | **0.002** |
| Any Complication | 1.20 | 1.01-1.42 | **0.036** |
| Vocal Cord Paralysis | 1.29 | 0.94-1.77 | 0.109 |
| Hoarseness | 1.15 | 0.66-2.00 | 0.611 |
| Hypocalcemia | 1.01 | 0.82-1.24 | 0.944 |
| Bleeding | 0.85 | 0.54-1.32 | 0.464 |
| Infection | 1.68 | 0.82-3.45 | 0.154 |
| Respiratory | 1.66 | 1.26-2.19 | **<0.001** |
| Acute Renal Failure | 1.87 | 1.13-3.09 | **0.015** |
| DVT/PE | 2.15 | 0.77-5.98 | 0.142 |
| Blood Transfusion | 1.86 | 0.87-3.98 | 0.111 |
| Wound Complication | 2.77 | 1.21-6.37 | **0.016** |
| ^a^ ^Adjusted for race/ethnicity, gender, income quartile by zip code, primary expected payer, admission type, procedure type, hospital bedsize, hospital location/teaching status and hospital region.^ | | | |

| **Supplemental Table 3**  Post-matching logistic regression analysis of morbidity in patients in non-obese group and the four obese subgroups stratified by BMI ranges undergoing thyroidectomy for thyroid cancer, National Inpatient Sample 2016-2020. | | | | |
| --- | --- | --- | --- | --- |
|  | BMI Subgroups | Adjusted Odds Ratio^a^ | 95%CI | P-value |
| Unfavorable Discharge | Group 1(BMI 30.0-34.9) | 1.02 | 0.66-1.57 | 0.927 |
|  | Group 2(BMI 35.0-39.9) | 1.47 | 0.96-2.25 | 0.073 |
|  | Group 3(BMI 40.0-44.9) | 2.01 | 1.32-3.05 | **0.001** |
|  | Group 4(BMI ≥45.0) | 1.58 | 1.02-2.45 | **0.040** |
| Any Complication | Group 1(BMI 30.0-34.9) | 1.09 | 0.84-1.42 | 0.522 |
|  | Group 2(BMI 35.0-39.9) | 1.29 | 0.99-1.69 | 0.064 |
|  | Group 3(BMI 40.0-44.9) | 1.10 | 0.81-1.48 | 0.543 |
|  | Group 4(BMI ≥45.0) | 1.13 | 0.84-1.53 | 0.405 |
| Vocal Cord Paralysis | Group 1(BMI 30.0-34.9) | 1.31 | 0.82-2.09 | 0.254 |
|  | Group 2(BMI 35.0-39.9) | 1.28 | 0.78-2.11 | 0.324 |
|  | Group 3(BMI 40.0-44.9) | 1.32 | 0.77-2.25 | 0.311 |
|  | Group 4(BMI ≥45.0) | 0.88 | 0.48-1.60 | 0.677 |
| Hoarseness | Group 1(BMI 30.0-34.9) | 1.52 | 0.71-3.23 | 0.283 |
|  | Group 2(BMI 35.0-39.9) | 0.81 | 0.30-2.15 | 0.666 |
|  | Group 3(BMI 40.0-44.9) | 0.74 | 0.25-2.16 | 0.576 |
|  | Group 4(BMI ≥45.0) | 1.53 | 0.67-3.52 | 0.313 |
| Hypocalcemia | Group 1(BMI 30.0-34.9) | 0.89 | 0.64-1.23 | 0.476 |
|  | Group 2(BMI 35.0-39.9) | 0.95 | 0.68-1.32 | 0.755 |
|  | Group 3(BMI 40.0-44.9) | 0.87 | 0.60-1.26 | 0.454 |
|  | Group 4(BMI ≥45.0) | 1.14 | 0.81-1.62 | 0.454 |
| Bleeding | Group 1(BMI 30.0-34.9) | 1.15 | 0.61-2.18 | 0.671 |
|  | Group 2(BMI 35.0-39.9) | 0.66 | 0.29-1.49 | 0.317 |
|  | Group 3(BMI 40.0-44.9) | 1.04 | 0.49-2.18 | 0.925 |
|  | Group 4(BMI ≥45.0) | 0.55 | 0.21-1.42 | 0.219 |
| Infection | Group 1(BMI 30.0-34.9) | 0.86 | 0.24-3.13 | 0.824 |
|  | Group 2(BMI 35.0-39.9) | 1.07 | 0.29-3.91 | 0.914 |
|  | Group 3(BMI 40.0-44.9) | 1.57 | 0.49-5.01 | 0.448 |
|  | Group 4(BMI ≥45.0) | 3.18 | 1.20-8.43 | **0.020** |
| Respiratory | Group 1(BMI 30.0-34.9) | 1.19 | 0.77-1.86 | 0.431 |
|  | Group 2(BMI 35.0-39.9) | 1.80 | 1.18-2.75 | **0.006** |
|  | Group 3(BMI 40.0-44.9) | 1.77 | 1.14-2.77 | **0.012** |
|  | Group 4(BMI ≥45.0) | 1.78 | 1.14-2.78 | **0.011** |
| Acute Renal Failure | Group 1(BMI 30.0-34.9) | 0.55 | 0.19-1.61 | 0.273 |
|  | Group 2(BMI 35.0-39.9) | 2.55 | 1.24-5.21 | **0.011** |
|  | Group 3(BMI 40.0-44.9) | 1.88 | 0.86-4.11 | 0.113 |
|  | Group 4(BMI ≥45.0) | 1.94 | 0.89-4.25 | 0.096 |
| DVT/PE | Group 1(BMI 30.0-34.9) | 1.40 | 0.26-7.38 | 0.695 |
|  | Group 2(BMI 35.0-39.9) | 0.80 | 0.09-7.13 | 0.843 |
|  | Group 3(BMI 40.0-44.9) | 3.83 | 0.97-15.15 | 0.056 |
|  | Group 4(BMI ≥45.0) | 2.41 | 0.55-10.51 | 0.242 |
| Blood Transfusion | Group 1(BMI 30.0-34.9) | 4.01 | 1.61-9.98 | **0.003** |
|  | Group 2(BMI 35.0-39.9) | 1.37 | 0.37-5.11 | 0.637 |
|  | Group 3(BMI 40.0-44.9) | 0.41 | 0.05-3.30 | 0.402 |
|  | Group 4(BMI ≥45.0) | 0.87 | 0.18-4.08 | 0.855 |
| Wound Complication | Group 1(BMI 30.0-34.9) | 4.06 | 1.52-10.87 | **0.005** |
|  | Group 2(BMI 35.0-39.9) | 1.04 | 0.21-5.06 | 0.961 |
|  | Group 3(BMI 40.0-44.9) | 0.76 | 0.09-6.17 | 0.794 |
|  | Group 4(BMI ≥45.0) | 3.18 | 0.99-10.21 | 0.051 |
| ^a^ ^Adjusted for race/ethnicity, gender, income quartile by zip code, primary expected payer, admission type, procedure type, hospital bedsize, hospital location/teaching status and hospital region.^ | | | | |
